# Efficacy and Safety of Adjusting Antimicrobial Therapy in Episodes of Fever and Neutropenia Catalogued as Fever of Unknown Origin in Children with Cancer: A Randomized Clinical Trial

**DOI:** 10.3390/antibiotics15080779

**Published:** 2026-08-13

**Authors:** Carolina Ibáñez, Romina Valenzuela, Marlon Barraza, Ana M. Álvarez, Ernesto Paya, Verónica Contardo, Valentina Gutiérrez, Karen Ducasse, Juan P. Torres, Paulina Coria, Verónica de la Maza, Daniela Martínez, Tamara Inostroza, María E. Santolaya

**Affiliations:** 1 Department of Pediatrics, Centro de Investigación Clínica Avanzada (CICA), Hospital Dr. Luis Calvo Mackenna, Faculty of Medicine, Universidad de Chile, Santiago 7500000, Chile; carolinaibanezg@uchile.cl (C.I.); mbarraza@calvomackenna.cl (M.B.);; 2 Department of Pediatrics, Hospital San Juan de Dios, Santiago 8320000, Chile; 3 Committee of Infectious Diseases, National Child Program of Antineoplastic Drugs (PINDA), Santiago 8320000, Chile; ernestopaya@uchile.cl (E.P.); vgutierrezu@uc.cl (V.G.);; 4 Department of Pediatrics, Hospital Dr. Exequiel González Cortés, Faculty of Medicine, Universidad de Chile, Santiago 8900000, Chile; 5 Department of Pediatrics, Hospital Dr. Roberto del Río, Faculty of Medicine, Universidad de Chile, Santiago 8380000, Chile; 6 Department of Pediatrics, Hospital Dr. Sótero del Río, Escuela de Medicina, Pontificia Universidad Católica, Santiago 8200000, Chile; 7 Department of Pediatrics, Hospital Gustavo Fricke, Viña del Mar 2520000, Chile; 8 Department of Pediatrics, Hospital San Borja Arriarán, Faculty of Medicine, Universidad de Chile, Santiago 8320000, Chile; 9 Department of Pediatrics, Hospital de Antofagasta, Antofagasta 1240000, Chile

**Keywords:** cancer, febrile neutropenia, stewardship, fever of unknown origin

## Abstract

**Background/Objectives:** The objective of this study is to evaluate the efficacy and safety of maintaining antimicrobial (AM) therapy or adjusting AM therapy during episodes of febrile neutropenia (FN) catalogued as a fever of unknown origin (FUO) in children with cancer. **Methods:** This is a prospective, multicenter, noninferiority, randomized study, approved by ethics committee, in children with episodes of FN in eight hospitals in Chile. Microbiological and molecular samples were drawn at admission. Patients with FUO (negative bacterial and viral study with no clinical focus suggesting infection) and favorable evolution during the first 48–72 h of AM therapy were randomized 1:1 to maintain or adjust treatment, reducing the number and spectrum of AMs. The primary endpoint was the percentage of episodes with an uneventful resolution; the secondary endpoints were the re-adjustment of AM therapy, days of fever/hospitalization/AM therapy, days of vancomycin and meropenem per 1000 days of neutropenia, number of AMs after randomization, pediatric intensive care unit (PICU) admission, sepsis, and death. **Results:** A total of 266 of 939 FN episodes recruited between March 2021 and January 2024 were catalogued as FUO, of which 231 had a favorable evolution at 48–72 h and were randomized, 111 to maintain and 120 to adjust AM therapy. Both groups presented the same percentage of uneventful resolution, with 106 (96%) in the group that maintain AM therapy and 114 (95%) in the adjusted group (*p* = 1.00); relative risk 1.01, (95%CI 0.95–1.06); absolute risk difference 0.01, (95%CI −0.05–0.06). **Conclusions:** The main finding of this study demonstrates that adjusting antimicrobial therapy appears safe in carefully selected FUO cases with a favorable early evolution, during episodes of FN catalogued as FUO in children with cancer. These findings open an opportunity to new AM stewardship strategies in this population, with a possible future impact on AM resistance.

## 1. Introduction

Febrile neutropenia (FN) is associated with high morbidity and mortality and is a common complication of antineoplastic treatment in children under cancer treatment, due to a greater predisposition to infections [1,2]. Children receiving chemotherapy experience between five to six episodes of FN over their period of treatment [1,2,3], requiring hospitalization, antimicrobial (AM) therapy, laboratory tests, imaging, and, eventually, Intensive Care Unit (ICU) admission [4,5].

Advances in diagnostic methods have made it possible to identify the etiology of infections in the context of FN, revealing that bacterial infections are responsible for 20–40% of cases, and respiratory viral infections about 30%, and 30–50% of FN episodes remain as a fever of unknown origin (FUO), without a bacterial nor respiratory viral identification that explains the cause of the fever [6,7]. The current guidelines for the management of children with cancer and FN episodes have established a standard of care for treatment that include the use of empirical AM therapy for every child with FN at the beginning of the episode based on risk stratification [2,4,8], with the corresponding stewardship adjustments after 48 or 72 h of evolution according to the clinical foci and microbiological findings, maintaining AM therapy in children with demonstrated or probable invasive bacterial infection and withholding AM therapy in children with viral respiratory infections. The present clinical recommendation for children with episodes of FN and FUO is to maintain broad-spectrum AM therapy for at least seven days [2,8]. The safety and efficacy of shortening these treatments or narrowing the spectrum of AM therapy has not been adequately studied, leaving a gap in knowledge [9], exposing patients to broad-spectrum AMs, favoring the selection of resistance strains.

In recent years, there has been a rise in AM resistance rates among children with cancer due to multi-drug-resistant (MDR) Gram-negative bacilli, attributed to risk factors such as a hospital stay over 48 h, the recent administration of empiric or targeted AMs, severe neutropenia, periods of neutropenia longer than 7 days and the use of ciprofloxacin prophylaxis [10,11,12,13]. On a recent report in a multinational cohort, including subjects from Australia, Brazil, Canada, Chile, Germany, Italy, Switzerland, and the Russian Federation, Castagnola et al. described 797 Gram-negative blood stream infections (BSIs) in pediatric patients undergoing chemotherapy or hematopoietic cell transplantation (HCT) in 13 centers worldwide, showing that the presence of resistant strains correlated with a higher risk of ICU admissions and a higher risk of death [14]. An analysis of AM resistance in isolates from children with episodes of FN in Chile from 2016 to 2021 showed that, among Gram-negative bacilli, there was 37% resistance to cefotaxime, 18% to piperacillin/tazobactam, 8% to amikacin, and 1.4% to carbapenems. This presents a challenging scenario due to the high mortality associated with these difficult-to-treat infections and the lack of new AM treatment alternatives to combat infections by MDR microorganisms [15].

The aim of this study was to evaluate the efficacy and safety of maintaining AM therapy or adjusted AM therapy during episodes of FN catalogued as FUO in children with cancer.

## 2. Results

### 2.1. Study Population

**Figure 1:**
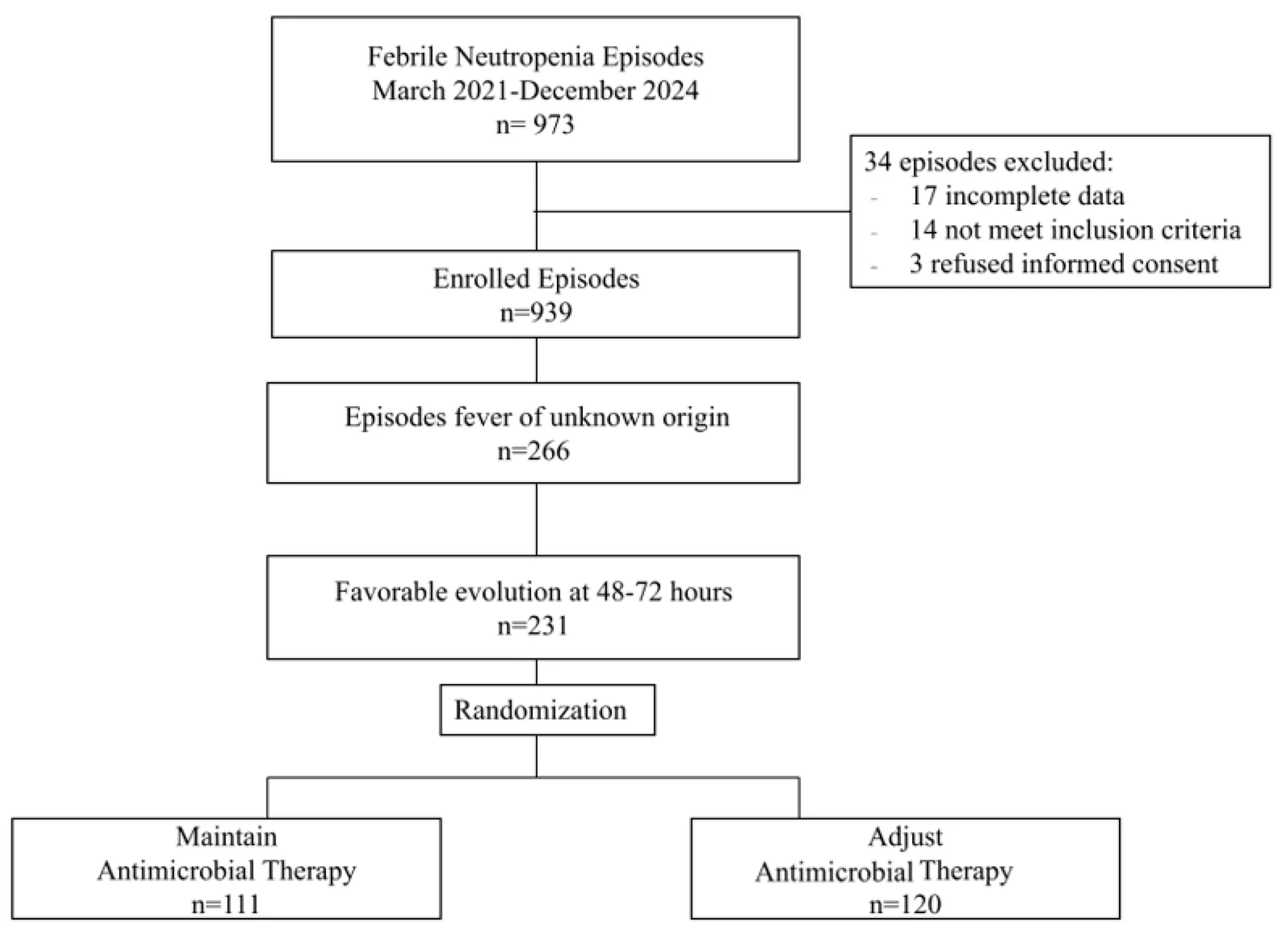
Flow of episodes of FN during the trial period.

In the study period, between March 2021 and January 2024, 973 episodes of FN were admitted and 939 episodes (97%) were enrolled. There were 34 excluded episodes, due to incomplete data (17), not meeting the inclusion criteria (14) and the rejection of informed consent (3). Among the 939 enrolled episodes, 266 (28%) were classified as FUO and 231 of them (86.8%) had a favorable evolution at 48–72 h of admission and were randomized as follows: 111 (48%) episodes to maintain AM therapy and 120 (52%) to adjust AM treatment (Figure 1).

The median age was 6 years (IQR 3–11) for both groups (*p* = 0.80); 55% and 54% were males, respectively (*p* = 1.00). The most frequent type of cancer was leukemia/lymphoma in 53% and 44% of children, maintained and adjusted, respectively (*p* = 0.19). The use of CVC was 97% for the group that maintained AM therapy and 98% for the adjusted group (*p* = 0.67). Episodes were categorized as high-risk febrile neutropenia (HRFN) in 69 (62%) and 64 episodes (53%) in the group that maintained and adjusted AM therapy, hypotension was present at admission in 1 episode (1%) in each group (*p* = 1.00), and there were no episodes of sepsis (Table 1).

### 2.2. Clinical Outcome

Children with FN episodes and FUO randomized to maintain or adjust AM therapy presented the same percentage of uneventful resolution, with 106 (96%) in the group that maintained AM therapy and 114 (95%) in the adjusted group (*p* = 1.00); relative risk 1.01, (95% CI 0.95–1.06); absolute risk difference 0.01, (95% CI −0.05–0.06). The re-adjustment of AM therapy was made in 5 (4%) of 120 episodes randomized to the adjusted AM therapy. There were five subjects that required the re–adjustment of antimicrobials after de-escalation, two subjects due to the re-appearance of fever, two due to an increase in CRP, and one due to both fever and CRP elevation. (Table 2). Children with adjusted AM therapy had less days of hospitalization, 7 days (5–8) versus 5 days, IQR (4–8) (*p* < 0.001), and less days of AM therapy, 6 (5–8) versus 5 (4–7) (*p* < 0.001). The median number of AMs after randomization was 2, IQR (1–2) in the group that maintain AM therapy versus 1, IQR (1–1) in the adjusted group, (*p* < 0.001). The number of days of vancomycin and meropenem per 1000 days of neutropenia showed a decrease in vancomycin uses in the adjusted group, from 93 to 59 days (*p* = 0.04). The percentage of PICU admission was 1% in the group that maintained AM therapy, and 2% in the adjusted group (*p* = 1.00); there were no cases of sepsis nor deaths in either group.

Two hundred and thirty-one episodes of FN were randomized, when analized by risk for invasive bacterial infection calculated at admission, 133 episodes were categorized as HRFN, 67 (97%) and 61 (95%) episodes had an uneventful resolution in the group that maintained or adjusted AM therapy, respectively (*p* = 0.67). For the 98 episodes of low-risk febrile neutropenia (LRFN), 39 (93%) and 53 (95%) of them had an uneventful resolution in both groups (*p* = 1.00).

## 3. Discussion

The main finding of this study demonstrates that adjusting the antimicrobial therapy appears safe in carefully selected FUO cases with a favorable early evolution during episodes of FN catalogued as FUO in children with cancer, founding the same percentage of children with an uneventful resolution, 96% in the group that maintained AM therapy compared to 95% in the adjusted group (*p* = 1.00); relative risk 1.01, (95%CI 0.95–1.06); absolute risk difference 0.01, (95%CI −0.05–0.06). Children in the adjusted group had fewer days of AM therapy, lower number of AMs after randomization, shorter hospital stays, and fewer days of vancomycin use per 1000 days of neutropenia.

The results of this study are consistent with those previously published by Clément et al. [8], following the 8th European Conference on Infections in Leukemia (ECIL-8) that recommend discontinuing empirical AM therapy in children with episodes of FN catalogued as FUO, after 72 h of treatment and at least 24–48 h of apyrexia [16], a guideline that is rarely applied in patients with high-risk FN. This was a retrospective study in a small number of patients, including a total of 51 FN episodes in which AM therapy could be discontinued in 19. Our results represent a step forward in this stewardship strategy, adding a prospective, multicenter, randomized, noninferiority study including 231 episodes of FN and FUO, randomized to maintain or adjust AM therapy. These two experiences, in two different regions of the world, represent an opportunity to continue advancing in this direction, especially considering that more than half of our patients were classified as high-risk upon admission to the hospital.

Despite the efforts to improve the rational AM management in children with cancer worldwide, we identify some gaps that need to be addressed in order to be able to face the increase in AM resistance in the population of immunocompromised children [17,18]. One of these gaps is trying to understand the meaning of a fever of unknown origin, in which, despite all efforts, no microbiological identification is obtained, and, sometimes, the AM treatment is prolonged for at least 7 days. The question is whether these children have a low-grade bacteremia not possible to identify with the current available automated blood cultures, that need at least 4 CFU/mL for detection, or that children with FUO have only an inflammatory response without infection [19]. In the first case, patients would require AM therapy, while, in the second case, they would not. Further research is needed to understand this clinical condition.

While researchers are trying to understand what FUO is, AM stewardship programs need to be implemented with several objectives, such as cost reduction, therapeutic optimization, and promoting actions that contribute to decreased AM resistance rates [20]. This study represents an AM stewardship proposal in children with cancer and episodes of FN and FUO with a favorable evolution, introducing the possibility to decrease the number of days of AM therapy and the length of the hospital stay, reducing the exposure to the risk of complications, like the acquisition of healthcare-associated infections (HAIs) secondary to multidrug-resistant microorganisms. This approach is consistent with international guidelines established by the Multinational Association for Supportive Care in cancer (MASCC), which advocates for the de-escalation or discontinuation of AM therapy in cases where no definitive source of infection can be identified [21].

Our study has limitations: the intervention was not double-blinded, a decision that was based on the fact that 97% and 98% of children in both groups received AM therapy through a central venous catheter, and we preferred to avoid the unnecessary manipulation using a placebo in children of the adjusted group, for the potential associated infections. A strength of the study is that we performed a prospective, multicenter, and randomized study with a significant sample size, providing a broad spectrum of patients.

More prospective, blinded trials, in different settings, may be needed before advancing further in this particular stewardship strategy.

## 4. Methods

### 4.1. Subjects

A randomized, multicenter, noninferiority trial was conducted between March 2021 and January 2024, funded by government grant, and conducted in 8 Chilean Hospitals, members of the PINDA (National Program of Anti Neoplastic Drugs) Network, namely, the following hospitals: Dr. Roberto del Río, San Juan de Dios, Sótero del Río, San Borja Arriarán, Gustavo Fricke, Base de Antofagasta, Dr. Exequiel González Cortés, and Dr. Luis Calvo Mackenna. Patients younger than eighteen years, in chemotherapy treatment for cancer, admitted for FN to any of the participating hospitals, were invited to participate and were enrolled after parental and child informed consent or assent (when older than 8 years of age). The study was approved by the Ethics Committee of each participating institution. Recipients of HCT were excluded.

### 4.2. Overall Study Design

Information regarding demographics was recorded: gender, age, type of cancer diagnosis, and hours of fever prior to admission. Moreover, clinical and laboratory information including general clinical assessment, axillary temperature (°C), blood pressure, complete blood count, and C-reactive protein (CRP). Microbiological and molecular study included blood cultures that were drawn from CVC and peripherally at the same time before starting AM therapy and molecular diagnostic evaluation (by multiplex-PCR) for respiratory pathogens through nasopharyngeal swabs.

Risk stratification of FN, based on the type of cancer, type/date of the last chemotherapy, blood pressure, platelet count, and CRP, per the Latin American Consensus and Guidelines for the Management of FN in children with cancer [2], as well as AM treatment, was indicated [2]. In cases with LRFN, treatment with a third-generation cephalosporin (ceftriaxone) was indicated, and, in children with HRFN, an anti-pseudomonal third-generation cephalosporin (ceftazidime) plus amikacin was prescribed with or without the addition of an anti-Gram-positive b-lactam or glycopeptide [2,22].

### 4.3. Definition of FUO and Randomization

A FN episode was catalogued as FUO if automated blood cultures, other sterile site cultures, and molecular panel for respiratory pathogens from nasopharyngeal sample obtained at admission were all negative, and subject did not have a clinical focus suggesting bacterial or viral infection.

After 48 or 72 h after admission, episodes were classified as having favorable or unfavorable evolution, defining favorable evolution as temperature <38.5 °C, clinical stability, no sepsis, nor new source or focus of infectious, and CRP less than 90 mg/L. Episodes of FN and FUO with favorable evolution were randomized 1:1 with the aid of a statistical software (GraphPad-Prism 9) to two groups, as described: maintain standard AM treatment or adjust AM treatment. The group that maintained AM therapy received AMs for at least 7 days either hospitalized or as outpatient, and the adjusted group reduced the number and spectrum of AMs either hospitalized or as outpatient; the AM choice was decided by the attending physician, with the aid of the trials infectious diseases specialist, mainly to ceftriaxone, cefotaxime, penicillin. and amikacin on intravenous alternatives, and. for oral, the choices were amoxicillin plus clavulanate, cefadroxil, cloxacillin, and ciprofloxacin. Both interventions, maintain and adjust, were discussed after randomization between the investigators and attending physicians caring for patients. Physical exam, laboratory including differential blood count, and CRP were done daily until discharge. Subjects in both groups were assessed daily until the defervescence and the recovery of the ANC to higher than 500/mm^3^, for at least 7 days.

### 4.4. Study Endpoints

The endpoints for this study were, primarily, the number (%) of episodes with uneventful resolution, and, secondarily, (i) re-adjustment of AM therapy, (ii) days of fever, (iii) number of days of hospitalization, (iv) number of days of AM therapy, (v) number of antimicrobials after randomization, (vi) number of days of AM therapy per vancomycin and meropenem per 1000 days of neutropenia, (vii) PICU admission, (viii) sepsis, and (ix) death.

A blinded investigator reviewed all case report forms at the end of the episodes and decided whether the resolution was uneventful or not (see definitions).

### 4.5. Laboratory Methods

BacT/ALERT, BioMérieux, Inc, Durham, North Carolina, USA or Bactec, BD Diagnostic Systems (a subsidiary of Becton, Dickinson and Company), Sparks, Maryland, USA, automated culture systems were used to process blood cultures (BCs), where positive BCs were stained for Gram determination and sub-cultured in specific agars, namely, MacConkey, chromogenic, and blood, and/or processed through automated methods such as MALDI-TOF, BioMérieux, Kioto, Japan and Manchester, UK or similar. Susceptibility determination was made through classic Kirby–Bauer and epsilometry in Mueller–Hinton in 5% sheep blood for *Streptococcus* spp. or by automated methods such as Vitek 2 BioMérieux, Inc, Hazelwood, Missouri / Durham, Noth Carolina, USA, Sensititre or others. Respiratory pathogen determination through nasopharyngeal swabs was performed with multiplex PCR (Film Array Respiratory-Panel^®^, BioFire Diagnostics (BioMérieux), LLC, Salt Lake City, Utah, USA) that includes 20 pathogens, in compliance with manufacturer’s instructions in addition to COVID-19 PCR.

### 4.6. Definitions

(a) Fever: temperature ≥38.5 °C in an axillary through single determination, or higher than 38 °C through 2 consecutive determinations one hour apart; (b) neutropenia: ANC ≤ 500/mm^3^; (c) HRFN: episode of FN with a risk factor, namely leukemia relapse or none lymphoblastic as cancer diagnosis, PCR of 90 mg/L o higher or hypotension, or both of the following risk factors: less than seven days between the onset of fever and the last chemotherapy and a platelet count ≤50,000/mm^3^; (d) LRFN: a FN episode without any of the risk factor for HRFN [2]; (e) invasive bacterial infection (IBI): bacterial blood stream infection (BSI) or bacterial isolation from a normally sterile site, or presence of clinical signs suggestive of localized bacterial infection with parenchymal involvement, with or without microbiological isolation; (f) viral respiratory tract infection (RTI): positive detection of one or more virus in respiratory sample; (g) sepsis: systemic inflammatory response syndrome in the presence of, or because of, suspected or proven infection, plus one of the following: cardiovascular organ dysfunction, or acute respiratory distress syndrome, or two or more other organ dysfunctions: neurologic, hematologic, renal, and hepatic [22]; (h) hypotension: blood pressure lower than the 5th percentile according to the age or less than 90/50 in children ≥10 years of age [23]; (i) episodes with favorable evolution, evaluated at 48–72 h after admission: temperature <38.5 °C, clinical stability, no sepsis nor new infectious foci, and CRP < 90 mg/L; (j) FUO: a FN episode was catalogued as FUO if automated blood cultures, other sterile site cultures, and molecular panels for respiratory pathogens from nasopharyngeal samples obtained at admission were all negative, and subject did not have a clinical focus suggesting bacterial or viral infection; (k) uneventful resolution: FN episode with favorable evolution determined by the randomized intervention and by the blind investigator when reviewing the episode at the end—for the group that maintained AM, therapy was established if no changes in the AM therapy were made and with no need of re-adjustment of the therapy in the group that had the intervention; and (l) mortality: death due to any cause during the follow up.

### 4.7. Statistical Analyses and Sample Size

In this noninferiority trial, the primary endpoint was the number and % of episodes with an uneventful resolution.

Sample size was calculated based on the hypothesis that the frequency of occurrence of uneventful resolution will be the same for both groups. Considering an uneventful outcome of 10% as previously reported for this population, a maximum acceptable difference of 10% between groups, a type I error of 0.05, and a potency of 90%, we calculated that 134 episodes were required for this intervention. *X*^2^ test or Fisher’s exact test were used for noncontinuous variables and t test or Mann–Whitney U-test for noncontinuous variables, per distribution. Calculation of relative risk and absolute risk difference for uneventful resolution between the exposed and unexposed with a 95% Cis interval was evaluated. All statistical analyses were performed with Stata SE14.0, Stata Corp 4905, USA.

## 5. Conclusions

In conclusion, according to our results, we suggest that it could be possible to adjust and shorten AM therapies in children with cancer and FN episodes catalogued as FUO that have a favorable evolution in the first 48–72 h of treatment. These findings open an opportunity to explore new diagnostic techniques to understand the etiology and significance of FUO, with the purpose of conducting future studies to determine the efficacy and safety of the withdrawal of AM therapy in this population, reducing the selective pressure of broad-spectrum AMs in children who do not need them.

## Figures and Tables

**Table 1:** Admission clinical characteristics of 231 episodes of fever and neutropenia with fever of unknown origin randomized to maintain or adjust antimicrobial therapy.

Characteristics at Admission	Type of Intervention		*p*
	Maintain AM Therapy	Adjust AM Therapy	
	*n* = 111 (48%)	*n* = 120 (52%)	
Age in years, median (IQR)	6 (3–11)	6 (3–11)	0.80
Gender (male), *n* (%)	61 (55)	65 (54)	1.00
Cancer diagnosis, *n* (%)			
Leukemia/Lymphoma	59 (53)	53 (44)	0.19
Leukemia relapse	14 (13)	9 (8)	0.27
Osteosarcoma	13 (12)	22 (18)	0.20
Other solid tumors *	25 (23)	36 (30)	0.23
Use of CVC, *n* (%)	108 (97)	118 (98)	0.67
Hours of fever prior to admission, median (IQR)	1 (1–2)	1 (1–2)	0.37
High-Risk Febrile Neutropenia, *n* (%)	69 (62)	64 (53)	0.19
Hypotension, *n* (%)	1 (1)	1 (1)	1.00
Sepsis, *n* (%)	0	0	

IQR: interquartile range, CVC: central venous catheter, AM: antimicrobial. * Other solid tumor: soft tissue sarcoma, neuroblastoma, Wilms tumor, Ewing sarcoma, retinoblastoma, hepatoblastoma, central nervous system tumors.

**Table 2:** Clinical outcome of 231 episodes of fever and neutropenia with fever of unknown origin in children with cancer, randomized to maintain or adjust antimicrobial therapy.

Clinical Outcome	Type of Intervention		*p*
	Maintain AM Therapy	Adjust AM Therapy	
	*n* = 111 (48%)	*n* = 120 (52%)	
Antimicrobial therapy adjustment, *n* (%)		120 (100)	
Uneventful resolution, *n* (%)	106 (96)	115 (96)	1.00
Re-adjustment of antimicrobial therapy, *n* (%)		5 (4)	
Days of fever, median (IQR)	2 (2–3)	2 (2–3)	0.82
Days of hospitalization, median (IQR)	7 (5–8)	5 (4–8)	<0.001
Days of antimicrobial treatment, median (IQR)	6 (5–8)	5 (4–7)	<0.001
Number of antimicrobials after randomization, median (IQR)	2 (1–2)	1 (1–1)	<0.001
Days of antimicrobial therapy per 1000 days of neutropenia			
Vancomycin	93	59	0.04
Meropenem	42	27	0.17
PICU Admission, *n* (%)	1 (1)	2 (2)	1.00
Sepsis, *n* (%)	0	0	
Death, *n* (%)	0	0	

IQR: interquartile range: AM: antimicrobial, PICU: Pediatric intensive care unit.

## Data Availability

Data supporting the reported results can be provided upon request.
